# Low copy number of the FCGR3B gene and rheumatoid arthritis: a case-control study and meta-analysis

**DOI:** 10.1186/ar3731

**Published:** 2012-02-07

**Authors:** Scott W Graf, Sue Lester, Johannes C Nossent, Catherine L Hill, Susanna M Proudman, Anita Lee, Maureen Rischmueller

**Affiliations:** 1 Department of Rheumatology, The Queen Elizabeth Hospital, 28 Woodville Rd, Woodville South, South Australia 5011, Australia; 2 Department of Rheumatology, Institute of Clinical Medicine, University of Tromsø 9037, Tromsø, Norway; 3 The Health Observatory, The University of Adelaide, Adelaide, South Australia 5005, Australia; 4 Rheumatology Unit, Royal Adelaide Hospital, North Terrace, Adelaide, South Australia 5000, Australia; 5 Discipline of Medicine, The University of Adelaide, Adelaide, South Australia 5005 Australia

## Abstract

**Introduction:**

Low copy number (CN) of the Fc gamma receptor 3B (*FCGR3B*) gene has been associated with systemic autoimmune disease. This receptor for IgG is present almost exclusively on neutrophils and plays a role in their interaction with immune complexes. At present the relationship between *FCGR3B *and rheumatoid arthritis (RA) is unclear. The aim of the present study was to investigate whether low CN of the *FCGR3B *gene is associated with susceptibility to RA.

**Method:**

The *FCGR3B *CN was determined using a custom Taqman^® ^CN assay (Hs04211858; Applied Biosystems, Foster City, CA, USA) in 197 RA patients, recruited from a tertiary setting, and in 162 population matched controls. Odds ratios for low CN (< 2) and high CN (> 2), both relative to the normal diploid 2CN, were estimated by logistic regression.

**Results:**

A significant association between RA and low *FCGR3B *CN was observed, with frequencies of 13.7% in RA patients compared with 6.2% in controls (odds ratio 2.5, 95% confidence interval 1.2 to 5.4, *P *= 0.017). No association was observed between low CN and the presence of rheumatoid factor, anti-cyclic citrullinated peptide antibodies or radiographic erosions in RA patients. A meta-analysis including six previous studies confirmed an association between RA and low *FCGR3B *CN (odds ratio 1.47, 95% confidence interval 1.13 to 1.92, *P *= 0.004).

**Conclusions:**

The present study confirms that a low CN of the *FCGR3B *gene is associated with susceptibility to RA. The association may be stronger in patients recruited from a tertiary setting, which may relate to disease severity and/or complications. The mechanism of susceptibility remains unclear and further study is required.

## Introduction

Variation in the copy number (CN) of genes within the human genome is an important source of genetic variation, and is defined as a sequence of DNA > 1 kb present in altered CN when compared with a reference genome [1]. In the diploid human genome, autosomal genes are normally present in two copies (one on each chromosome). When copy number variation (CNV) is present, however, the number of copies can vary from zero to greater than two. Studies have demonstrated a large number of genes within the human genome that display CNV in a relatively high frequency. CNV may be a major source of quantitative variation in expression, and has been proposed to contribute to phenotypic diversity and disease susceptibility [2-4]. There are increasing reports of associations between CNV of certain genes, coding for various components of the immune system, and autoimmune diseases such as Crohn's disease [5], rheumatoid arthritis (RA) [6], systemic lupus erythematosis (SLE) [7], Sjögren's syndrome [8], and psoriasis [9].

Fc gamma receptors are present predominantly on myeloid cells and interact with the Fc portion of the IgG molecule. They can be either stimulatory or inhibitory and play an integral role in the identification and destruction of both endogenous and foreign opsonised material. A number of subtypes have been identified with extensive structural diversity leading to differences in binding capacity, signal transduction pathways and cell-type surface expression [10]. The Fc gamma receptor 3B (*FCGR3B*) gene is a stimulatory receptor that is present almost exclusively on the surface of neutrophils. Its precise function in the immune system remains to be clarified but there is accumulating evidence that it plays a role in the interaction between immune complexes and neutrophils [11-13]. CNV has been well characterised in the Fc gamma receptor region (Chr 1q23-24) [14], and a low CN of *FCGR3B *has been associated with SLE and Sjögren's syndrome [7,15,16] as well as with RA [17]. The evidence to date for an association between low CN and RA has been contradictory, with studies showing both the presence and absence of a significant relationship [15,17,18]. The aim of our study was to determine whether there is an association between CN of the *FCGR3B *gene and RA, compared with population controls. The identification of such a relationship and confirmation of a low CN of *FCGR3B *as a susceptibility factor would contribute to our evolving understanding of the pathogenesis of RA.

## Materials and methods

### Study subjects

The study sample included 197 patients recruited from the rheumatology outpatient services at The Queen Elizabeth Hospital and the Royal Adelaide Hospital, two tertiary-level hospitals in South Australia. All subjects had been diagnosed with RA according to the 1987 American College of Rheumatology criteria for RA [19]. Recruitment of patients and DNA extraction occurred over the period of 2006 to 2011. Clinical characteristics including gender, rheumatoid factor (RF) and anti-cyclic citrullinated peptide (anti-CCP) antibody status and the presence or absence of erosive disease were collected. The presence of erosive disease was defined on the basis of radiographs, formally reported by a radiologist, showing evidence of erosions, at any articular site, consistent with a diagnosis of RA. The designation of no erosive disease required formal bilateral hand and feet radiographs reported as showing no evidence of erosions. Adequate radiographic data were available in 139 patients (71%). The control group included 162 population-based Caucasian healthy subjects (53% female, median age 56 years).

The study was conducted in accordance with the Declaration of Helsinki and approved by the Central North Adelaide Health Service Ethics of Human Research Committee. All participants provided informed, written consent.

### Determination of *FCGR3B *copy number

The *FCGR3B *CN was determined from genomic DNA using a custom Taqman^® ^CN real-time, quantitative PCR assay (Hs04211858, FAM-MGB dual-labelled probe; Applied Biosystems, Foster City, CA, USA) with RNase P (4403326, VIC-TAMRA dual-labelled probe; Applied Biosystems) as the reference assay, performed as a duplex reaction. The assay was performed according to the manufacturer's instructions and quantitative PCR reactions were run on an Applied Biosystems 7300 Real Time PCR machine. All samples were tested in triplicate, and fluorescence signals were normalised to ROX.

The TaqMan^® ^quantitative PCR assay was initially validated against an alternative, end-point PCR paralogue ratio (single primer pair) assay, which estimates *FCGR2C *CN relative to *FCGR2A*, as previously described [16]. The rationale for this is that several studies have reported complete agreement (that is, linkage disequilibrium) between *FCGR2C *CN and *FCGR3B *CN [14,16]. The amount of PCR product corresponding to each gene was quantified by measuring respective peak heights in the QIAxel capillary electrophoresis system (Qiagen, Hilden, Germany), and CN assignments were performed following cluster analysis of the log ratio of the peak heights. We tested 100 samples in parallel, and following independent analysis there was complete concordance in CN assignment for 99 of these samples; for the remaining sample, CN could not be unequivocally assigned by the paralogue ratio assay results, and was not retested.

The TaqMan^® ^assay quantitative PCR amplification curves were analysed using three different quantitation points (Cq): Ct (autobaseline), 0.2 cycle threshold, as recommended by the manufacturer); Cy0, determined from a five-parameter log-logistic curve analysis from the ΔRn data [20]; and CpD1, derived from a six-parameter log-logistic curve (which explicitly models the baseline) from the Rn data. Cy0 and CpD1 estimates were obtained using the R project statistical computing qpcR library [21,22]. Analysis was performed on a plate-by plate basis, and the CN was assigned from the raw Cq values using CopyCaller™ software (version 1.0; Applied Biosystems). This software employs a clustering algorithm and assigns the cluster with the most samples as 2CN. Reference samples of 1CN, 2CN and 3CN (validated using the paralogue ratio assay) were included on each plate to ensure that the most frequent CN categories were represented on each plate, and to confirm consistency in correct CN assignment between plates.

The CopyCaller™ software also provides extensive diagnostics for the validity of the results, which were accepted only when confidence in discrete CN assignment was > 80%, the standard deviation of the sample replicate ΔCq estimates was < 0.20, and a reference gene Cq was < 32. CN assignment for results that failed any of these criteria was unequivocally resolved by retesting. Although different, all three Cq estimates gave discrete and identical CN assignments. An occasional spuriously high CN (> 5) result was obtained via the recommended Ct method however, due to sporadic FAM auto-baselining problems. We observed no discrepancies with the Cy0 method. This method has been reported to overcome quantitation inaccuracy in the presence of amplification inhibition [20] so it is therefore unlikely that variability in DNA quality was a confounder in CN assignment, as has been previously reported [17]. The CpD1 estimate (from unbaselined data) consistently resulted in the lowest standard deviation for replicate ΔCq estimates. Interestingly, the CpD1 estimate from baselined data did not perform well in terms of discrete CN assignments (nor did the CpD2 estimate from either baselined or unbaselined data). Both baselining and Cq estimation algorithms, which differ between instrumentation and are generally not reported, can therefore substantially influence the performance of the assay.

**Figure 1:**
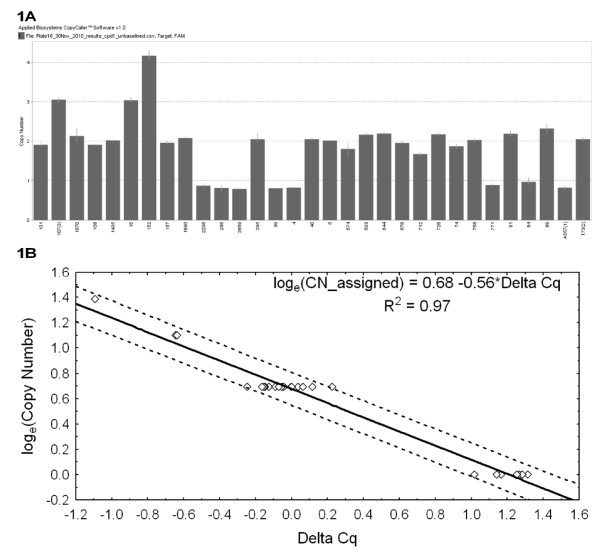
**Analysis summary for a typical TaqMan^® ^Fc gamma receptor 3B copy number assay plate**. Analysis summary for a typical TaqMan^® ^Fc gamma receptor 3B (*FCGR3B*) gene copy number (CN) assay plate with one, two, three, and four CN samples using CpD1 quantitation point (Cq) estimates. **(A) **Bar chart output from the CopyCaller™ analysis software demonstrating that the continuous estimated CN ratios for each sample form distinct clusters with values that are very close to integer values. Three reference samples are included on the plate. The 3CN reference sample is second from the left, and the 1CN and 2CN reference samples are the second last and last (right), respectively. The overall estimate of the standard deviation for replicate ΔCq values for this plate was 0.07. **(B) **Scatter plot of the ΔCq values versus the (natural) log of the assigned CN. The straight line represents the fitted regression line and the dotted lines represent the 95% prediction intervals for the regression. The intercept term for the regression analysis may vary between plates due to small differences in the pipetting of reagents. CN assignment must therefore be performed on an individual plate basis.

Overall, our methodology resulted in clear assignment of a discrete *FCGR3B *CN to each sample, as is evident from the results for a sample plate depicted in Figure 1.

### Statistical analysis

The distributions of *FCGR3B *CN between patients and controls and between patients with and without RF, anti-CCP and radiographic erosions were compared using the maximum likelihood chi-square test with two degrees of freedom. Logistic regression analysis was used to calculate odds ratios (ORs) for low CN (< 2) and high CN (> 2), both relative to the normal diploid 2CN for RA patients compared with controls. *P *< 0.05 was considered statistically significant. Analysis was performed using Statistica version 6 (Statsoft, Tulsa, OK, USA).

A random-effects meta-analysis of previously published studies examining the relationship between low CN of *FCGR3B *and RA was performed using the R metafor library [21,23] and the restricted maximum-likelihood method.

## Results

Of the 197 RA patients, 145 (74%) were female. Of those for whom autoantibody status was determined, 136 (75%) were RF-positive and 68 (60%) were anti-CCP-positive. Of those with adequate radiographic data, 96 (69%) had erosive disease.

The majority of patients in both groups had two copies of the *FCGR3B *gene (75.6% in the RA cohort and 85.8% in the controls). A higher frequency of low *FCGR3B *CN (< 2) was observed in the RA patients compared with controls (13.7% and 6.2%, respectively), with OR 2.5 (95% confidence interval 1.2 to 5.4, *P *= 0.017) indicating an increased susceptibility for RA being associated with a low CN. No significant difference was seen in the frequency of high CN (> 2) between RA patients and controls (10.7% and 8%, respectively; OR 1.5, 95% confidence interval 0.7 to 3.1, *P *= 0.27) (Table 1).

**Table 1:** Frequency of FCGR3B copy number variants in rheumatoid arthritis patients compared with controls

FCGR3B CN	RA (*n *= 197)	Controls (*n *= 162)	Odds ratio (95% CI)	*P *value
< 2	27 (13.7%)	10 (6.2%)	2.5 (1.2 to 5.4)	0.017
2	149 (75.6%)	139 (85.8%)	1	
> 2	21 (10.7%)	13 (8.0%)	1.5 (0.7 to 3.1)	0.27
Global test	χ^2 ^= 6.9, degrees of freedom = 2, *P *= 0.031			

Data presented as *n *(%). CI, confidence interval; CN, copy number; FCGR3B, Fc gamma receptor 3B; RA, rheumatoid arthritis.

No significant relationships were observed between *FCGR3B *CN and RF, anti-CCP antibodies (Table 2) or radiographic erosions (data not shown) within RA patients.

**Table 2:** Comparative frequency of FCGR3B copy number variants with rheumatoid factor and anti-cyclic citrullinated peptide autoantibodies

FCGR3B CN	Rheumatoid factor		Anti-CCP	
	
	Positive	Negative	Positive	Negative
< 2	19 (13.97%)	5 (11.11%)	7 (10.29%)	5 (10.87%)
2	106 (77.94%)	33 (73.33%)	52 (76.47%)	36 (78.26%)
> 2	11 (8.09%)	7 (15.56%)	9 (13.24%)	5 (10.87%)
Global test	χ^2 ^= 2.03, degrees of freedom = 2, *P *= 0.363		χ^2 ^= 0.15, degrees of freedom = 2, *P *= 0.93	

Comparative frequency of Fc gamma receptor 3B (FCGR3B) copy number (CN) variants in rheumatoid arthritis patients positive and negative for rheumatoid factor and anti-cyclic citrullinated peptide (anti-CCP) autoantibodies. Data presented as *n *(%).

**Figure 2:**
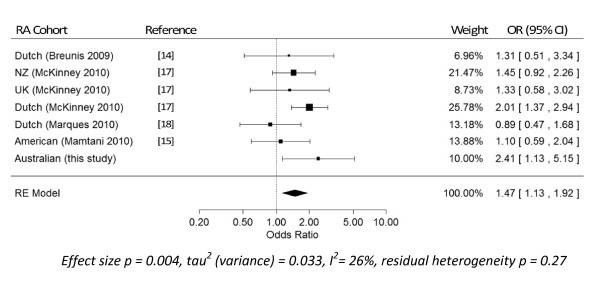
**Association of low Fc gamma receptor 3B copy number with rheumatoid arthritis**. Random effects (RE) meta-analysis of the association of low Fc gamma receptor 3B (FCGR3B) copy number (CN < 2) versus FCGR3B CN ≥ 2 with rheumatoid arthritis (RA). CI, confidence interval; NZ, New Zealand; OR, odds ratio.

An update of the meta-analysis for the association of low *FCGR3B *CN (< 2) with RA, originally reported by McKinney and colleagues [17], was also performed, which included two subsequently published studies [15,18] in addition to the current study. The meta-analysis now includes data from seven RA cohorts, predominantly of Northern European Caucasians, totalling 2,475 RA patients and 2,320 controls. This meta-analysis confirms that low *FCGR3B *CN is a risk factor for RA (OR 1.47, 95% confidence interval 1.13 to 1.92, *P *= 0.004, random-effects estimate), even though many individual studies failed to reach statistical significance (Figure 2). Further, the *I*^2 ^value of 26% indicates that the studies are relatively homogeneous.

## Discussion

Low *FCGR3B *CN, while best characterised in SLE, has been associated with a range of systemic autoimmune diseases [7]. In our study we have demonstrated a significant association between low CN of the *FCGR3B *gene and RA. Further, we have confirmed this association with a meta-analysis that includes seven published patient cohorts; while the association is relatively modest and somewhat less than that reported for SLE (OR 1.47, 95% confidence interval 1.13 to 1.92), it is highly significant (*P *= 0.004). This relatively modest effect size may explain why individual, smaller studies have reported inconsistent results.

We observed a relatively high effect size in our study. Our RA patients were recruited from rheumatology outpatient services in two large metropolitan tertiary centres, and therefore our cohort may be biased towards a more severe, or refractory, disease phenotype, which is reflected in the high incidence of seropositivity and erosive disease. However, the association between low *FCGR3B *CN and disease severity, or response to therapy, remains to be evaluated.

Only one previous study has examined the relationship between seropositivity and *FCGR3B *CN. McKinney and colleagues observed a higher frequency of low CN in RF-negative patients, but this was not observed in all cohorts. The anti-CCP status was available in only one cohort and its relationship with CN was not analysed [17]. In our patient group, we found no evidence for an association between *FCGR3B *low CN and RF or anti-CCP status nor the presence of erosive disease.

If the presence of a low *FCGR3B *CN increases the susceptibility to RA then the function of this receptor and how a low CN predisposes to the development of autoimmune disease needs to be considered. Fc gamma receptors can be either stimulatory or inhibitory in terms of the effect the binding of IgG has on the effector cell. FCGR3B is a stimulatory receptor that is located predominately on the cell surface of neutrophils [10].

The function of *FCGR3B *in the immune response has been studied by a number of groups, with all evidence suggesting this receptor plays a role in the interaction between neutrophils and immune complexes [11,12]. Would a low genomic CN of the *FCGR3B *gene have an effect on this? Willcocks and colleagues demonstrated a clear correlation between CN and FCGR3B cell surface expression, neutrophil adherence to IgG-coated surfaces and immune complex uptake [13], suggesting that low CN of the *FCGR3B *gene has physiological implications in the neutrophil-immune complex interaction.

If *FCGR3B *plays a role in the adherence of neutrophils to immune complexes and their subsequent clearance, then by what mechanism could a reduced CN predispose to autoimmune disease? It is possible that persistent immune complexes, due to ineffective clearance, have the potential for promoting or exacerbating autoimmunity. The lack of an association between low *FCGR3B *CN and RF or anti-CCP in RA patients, however, suggests that this potentially impaired interaction may not specifically involve autoantigen-immune complexes. Another possibility is that an impaired response to an infectious agent and delayed clearance could promote the breakdown of self-tolerance and the subsequent development of autoimmunity. There is some evidence of a relationship between *FCGR3B *and susceptibility to infection to support this theory, but the data are contradictory [16,24-26].

A reduction in *FCGR3B *density on the surface of neutrophils could disturb a delicate balance with the subsequent dominance of other Fc gamma receptors with greater stimulatory activity and potential pathogenicity. A study by Tsuboi and colleagues examined and compared the function of *FCGR2A *and *FCGR3B*, both stimulatory Fc gamma receptors [27]. Transgenic mice expressing human *FCGR2A *and/or *FCGR3B *on neutrophils were challenged with immune complexes. Compared with neutrophils expressing *FCGR3B*, neutrophils expressing *FCGR2A *demonstrated significantly greater adhesion to immune complexes and release of reactive oxygen species. The mice with neutrophils expressing *FCGR2A *developed an autoimmune nephritis with the accumulation of glomerular neutrophils, interstitial macrophages and CD4^+ ^T cells, the development of renal dysfunction and death. Clinical evidence of renal disease was absent in *FCGR3B*-deficient mice, with histology showing the accumulation of glomerular neutrophils with minimal interstitial leukocytic infiltrate and no evidence of renal injury. These results suggest that *FCGR3B *acts as a benign immune complex clearance mechanism and that reduced expression may lead to increased *FCGR2A*-immune complex interaction and thus promote immune complex-mediated tissue damage. Further support for this theory comes from evidence that immune complexes containing citrullinated proteins are potent stimulators of TNF production by macrophages via *FCGR2A *and are critical for the development of immune-complex-mediated inflammatory arthritis in murine models [28-30].

A final possibility is that *FCGR3B *CN is in linkage disequilibrium with another functional variant in the Fc gamma receptor region. For example, *FCGR3B *is adjacent to *FCGR2C *and the CNV of these two genes is in complete linkage disequilibrium [31].

In the present study we have demonstrated that a low CN of the *FCGR3B *gene is associated with RA. This raises the question of what role a low CN may play in the disease phenotype. The fact that we were able to demonstrate a significant relationship in our cohort of patients from a tertiary referral centre suggests that low CN may be associated with more severe or refractory disease. Future research should explore these relationships further as well as consider whether low FCGR3B CN increases the risk of infection in the context of immunosuppressive disease-modifying therapy.

## Conclusions

While previous studies have reported inconsistent results, the present study finds that a low CN of the *FCGR3B *gene is associated with susceptibility to RA. The mechanism of this increased susceptibility remains unclear, although there are a number of potential possibilities based upon the growing understanding of the function of the FCGR3B receptor. Such knowledge could potentially contribute to our understanding of the complex pathogenesis of RA.

## Abbreviations

anti-CCP: anti-cyclic citrullinated peptide; Cq: quantitation points; CN: copy number; CNV: copy number variation; FCGR3B: Fc gamma receptor 3B; OR: odds ratio; PCR: polymerase chain reaction; RA: rheumatoid arthritis; RF: rheumatoid factor; SLE: systemic lupus erythematosis.

## Competing interests

The authors declare that they have no competing interests.

## Authors' contributions

SWG participated in the acquisition of data, interpretation of results and review of the literature, and drafted the manuscript. SL participated in the design and coordination of the study, acquisition of data and interpretation of results, and assisted with drafting the manuscript. HN participated in the design and coordination of the study and the acquisition of data. CLH, SP and AL participated in the acquisition of clinical samples and clinical data and assisted with drafting the manuscript. MR conceived of the study and participated in its design and coordination and assisted with the acquisition of data. All authors read and approved the final manuscript.

## References

[B1] Feuk L Carson AR Scherer SW Structural variation in the human genome Nat Rev Genet 2006 7 85 97 16418744 10.1038/nrg1767

[B2] Redon R Ishikawa S Fitch KR Feuk L Perry GH Andrews TD Fiegler H Shapero MH Carson AR Chen W Cho EK Dallaire S Freeman JL González JR Gratacòs M Huang J Kalaitzopoulos D Komura D MacDonald JR Marshall CR Mei R Montgomery L Nishimura K Okamura K Shen F Somerville MJ Tchinda J Valsesia A Woodwark C Yang F Global variation in copy number in the human genome Nature 2006 444 444 454 10.1038/nature05329 17122850 PMC2669898

[B3] Sebat J Lakshmi B Troge J Alexander J Young J Lundin P Månér S Massa H Walker M Chi M Navin N Lucito R Healy J Hicks J Ye K Reiner A Gilliam TC Trask B Patterson N Zetterberg A Wigler M Large-scale copy number polymorphism in the human genome Science 2004 305 525 528 10.1126/science.1098918 15273396

[B4] Wong KK deLeeuw RJ Dosanjh NS Kimm LR Cheng Z Horsman DE MacAulay C Ng RT Brown CJ Eichler EE Lam WL A comprehensive analysis of common copy-number variations in the human genome Am J Hum Genet 2007 80 91 104 10.1086/510560 17160897 PMC1785303

[B5] Bentley RW Pearson J Gearry RB Barclay ML McKinney C Merriman TR Roberts RL Association of higher DEFB4 genomic copy number with Crohn's disease Am J Gastroenterol 2010 105 354 359 10.1038/ajg.2009.582 19809410

[B6] McKinney C Merriman ME Chapman PT Gow PJ Harrison AA Highton J Jones PB McLean L O'Donnell JL Pokorny V Spellerberg M Stamp LK Willis J Steer S Merriman TR Evidence for an influence of chemokine ligand 3-like 1 (CCL3L1) gene copy number on susceptibility to rheumatoid arthritis Ann Rheum Dis 2008 67 409 413 17604289 10.1136/ard.2007.075028

[B7] Fanciulli M Norsworthy PJ Petretto E Dong R Harper L Kamesh L Heward JM Gough SC de Smith A Blakemore AI Froguel P Owen CJ Pearce SH Teixeira L Guillevin L Graham DS Pusey CD Cook HT Vyse TJ Aitman TJ FCGR3B copy number variation is associated with susceptibility to systemic, but not organ-specific, autoimmunity Nat Genet 2007 39 721 723 10.1038/ng2046 17529978 PMC2742197

[B8] Mamtani M Rovin B Brey R Camargo JF Kulkarni H Herrera M Correa P Holliday S Anaya JM Ahuja SK CCL3L1 gene-containing segmental duplications and polymorphisms in CCR5 affect risk of systemic lupus erythaematosus Ann Rheum Dis 2008 67 1076 1083 10.1136/ard.2007.078048 17971457 PMC3786698

[B9] Hollox EJ Huffmeier U Zeeuwen PL Palla R Lascorz J Rodijk-Olthuis D van de Kerkhof PC Traupe H de Jongh G den Heijer M Reis A Armour JA Schalkwijk J Psoriasis is associated with increased beta-defensin genomic copy number Nat Genet 2008 40 23 25 10.1038/ng.2007.48 18059266 PMC2447885

[B10] Salmon JE Pricop L Human receptors for immunoglobulin G: key elements in the pathogenesis of rheumatic disease Arthritis Rheum 2001 44 739 750 10.1002/1529-0131(200104)44:4<739::AID-ANR129>3.0.CO;2-O 11315912

[B11] Coxon A Cullere X Knight S Sethi S Wakelin MW Stavrakis G Luscinskas FW Mayadas TN Fc gamma RIII mediates neutrophil recruitment to immune complexes. A mechanism for neutrophil accumulation in immune-mediated inflammation Immunity 2001 14 693 704 10.1016/S1074-7613(01)00150-9 11420040

[B12] Fossati G Moots RJ Bucknall RC Edwards SW Differential role of neutrophil Fcgamma receptor IIIB (CD16) in phagocytosis, bacterial killing, and responses to immune complexes Arthritis Rheum 2002 46 1351 1361 10.1002/art.10230 12115243

[B13] Willcocks LC Lyons PA Clatworthy MR Robinson JI Yang W Newland SA Plagnol V McGovern NN Condliffe AM Chilvers ER Adu D Jolly EC Watts R Lau YL Morgan AW Nash G Smith KG Copy number of FCGR3B, which is associated with systemic lupus erythematosus, correlates with protein expression and immune complex uptake J Exp Med 2008 205 1573 1582 10.1084/jem.20072413 18559452 PMC2442635

[B14] Breunis WB van Mirre E Geissler J Laddach N Wolbink G van der Schoot E de Haas M de Boer M Roos D Kuijpers TW Copy number variation at the FCGR locus includes FCGR3A, FCGR2C and FCGR3B but not FCGR2A and FCGR2B Hum Mutat 2009 30 E640 E650 10.1002/humu.20997 19309690

[B15] Mamtani M Anaya JM He W Ahuja SK Association of copy number variation in the FCGR3B gene with risk of autoimmune diseases Genes Immun 2010 11 155 160 10.1038/gene.2009.71 19741716

[B16] Niederer HA Willcocks LC Rayner TF Yang W Lau YL Williams TN Scott JA Urban BC Peshu N Dunstan SJ Hien TT Phu NH Padyukov L Gunnarsson I Svenungsson E Savage CO Watts RA Lyons PA Clayton DG Smith KG Copy number, linkage disequilibrium and disease association in the FCGR locus Hum Mol Genet 2010 19 3282 3294 10.1093/hmg/ddq216 20508037 PMC2908468

[B17] McKinney C Fanciulli M Merriman ME Phipps-Green A Alizadeh BZ Koeleman BP Dalbeth N Gow PJ Harrison AA Highton J Jones PB Stamp LK Steer S Barrera P Coenen MJ Franke B van Riel PL Vyse TJ Aitman TJ Radstake TR Merriman TR Association of variation in Fcgamma receptor 3B gene copy number with rheumatoid arthritis in Caucasian samples Ann Rheum Dis 2010 69 1711 1716 10.1136/ard.2009.123588 20472591 PMC3670580

[B18] Marques RB Thabet MM White SJ Houwing-Duistermaat JJ Bakker AM Hendriks GJ Zhernakova A Huizinga TW van der Helm-van Mil AH Toes RE Genetic variation of the Fc gamma receptor 3B gene and association with rheumatoid arthritis PLoS One 2010 5 e13173 10.1371/journal.pone.0013173 20957197 PMC2950138

[B19] Arnett FC Edworthy SM Bloch DA McShane DJ Fries JF Cooper NS Healey LA Kaplan SR Liang MH Luthra HS The American Rheumatism Association 1987 revised criteria for the classification of rheumatoid arthritis Arthritis Rheum 1988 31 315 324 10.1002/art.1780310302 3358796

[B20] Guescini M Sisti D Rocchi MB Stocchi L Stocchi V A new real-time PCR method to overcome significant quantitative inaccuracy due to slight amplification inhibition BMC Bioinformatics 2008 9 326 10.1186/1471-2105-9-326 18667053 PMC2533027

[B21] R Development Core Team R: A Language and Environment for Statistical Computing 2011 Vienna: R Foundation for Statistical Computing

[B22] Ritz C Spiess AN qpcR: an R package for sigmoidal model selection in quantitative real-time polymerase chain reaction analysis Bioinformatics 2008 24 1549 1551 10.1093/bioinformatics/btn227 18482995

[B23] Viechtbauer W Conducting meta-analyses in R with the metafor package J Stat Software 2010 36 1 48

[B24] Fu Y Korostoff JM Fine DH Wilson ME Fc gamma receptor genes as risk markers for localized aggressive periodontitis in African-Americans J Periodontol 2002 73 517 523 10.1902/jop.2002.73.5.517 12027254

[B25] Hughes LB Criswell LA Beasley TM Edberg JC Kimberly RP Moreland LW Seldin MF Bridges SL Genetic risk factors for infection in patients with early rheumatoid arthritis Genes Immun 2004 5 641 647 10.1038/sj.gene.6364137 15526004

[B26] Omi K Ohashi J Patarapotikul J Hananantachai H Naka I Looareesuwan S Tokunaga K Fcgamma receptor IIA and IIIB polymorphisms are associated with susceptibility to cerebral malaria Parasitol Int 2002 51 361 366 10.1016/S1383-5769(02)00040-5 12421634

[B27] Tsuboi N Asano K Lauterbach M Mayadas TN Human neutrophil Fcgamma receptors initiate and play specialized nonredundant roles in antibody-mediated inflammatory diseases Immunity 2008 28 833 846 10.1016/j.immuni.2008.04.013 18538590 PMC2577844

[B28] Clavel C Nogueira L Laurent L Iobagiu C Vincent C Sebbag M Serre G Induction of macrophage secretion of tumor necrosis factor alpha through Fcgamma receptor IIa engagement by rheumatoid arthritis-specific autoantibodies to citrullinated proteins complexed with fibrinogen Arthritis Rheum 2008 58 678 688 10.1002/art.23284 18311806

[B29] Sokolove J Zhao X Chandra PE Robinson WH Immune complexes containing citrullinated fibrinogen costimulate macrophages via Toll-like receptor 4 and Fcgamma receptor Arthritis Rheum 2011 63 53 62 20954191 10.1002/art.30081 PMC3015008

[B30] Tsuboi N Ernandez T Li X Nishi H Cullere X Mekala D Hazen M Köhl J Lee DM Mayadas TN Regulation of human neutrophil Fcgamma receptor IIa by C5a receptor promotes inflammatory arthritis in mice Arthritis Rheum 2011 63 467 478 10.1002/art.30141 21280001 PMC3128197

[B31] Breunis WB van Mirre E Bruin M Geissler J de Boer M Peters M Roos D de Haas M Koene HR Kuijpers TW Copy number variation of the activating FCGR2C gene predisposes to idiopathic thrombocytopenic purpura Blood 2008 111 1029 1038 17827395 10.1182/blood-2007-03-079913

